# Incidents of Animal Life

**Published:** 1886-12-25

**Authors:** F. O. Morris

**Affiliations:** Rector of Nunburnholme, Yorkshire; author of "British Birds," etc.


### Incidents of Animal Life.

Collated by the Rev. F. O. MORRIS, B.A.,

Rector of Nunburnholme, Yorkshire; author of "British Birds," etc.

> "He prayeth best, who loveth best
> All things both great and small;
> For the dear God who loveth us,
> He made and loveth all."—Coleridge.

### Stories about Rats.

Rats and their Ways.

"I have read with much interest various letters in *The Times* respecting the rat. The account, however, given by some of the correspondents of the method adopted by these animals for taking eggs is not new. I have certainly read it before, and it is referred to in an article on rats in the *Quarterly Review* of Jan. 1857, vol. 101. If my readers have not seen that article, perhaps they will pardon me for drawing their attention to it, though it may be that they have read it many years ago. The theory of co-operative action on the part of rats would, if true, account for a circumstance which came under my own observation, but which is otherwise inexplicable. When quite a young man, I was left in charge of a large old-fashioned house in the city of Bristol. The servants, the only other inmates, were to bring some keys to me every night, and to come for them again in the morning. The possession of these keys was (for reasons I need not specify) a serious responsibility, and for security I used to place them in the top drawer of a chest near my bedside. The room in which I slept was far away from any other inhabited one, and I never went to bed without a feeling of nervousness and anxiety. Many a time I lay awake conjuring up all kinds of fearful contingencies, and wishing my term of responsibility was over."

A Burglarious Combination.

"One night I was startled from a perturbed sleep by a rattling of my bunch of keys, clear and unmistakable. I looked up; the chest of drawers was between me and a window, an old-fashioned leaded casement, and the moonlight was shining brightly upon the top of it. In this light and over the top of this chest, and reaching into the drawers where the keys were deposited, I saw what I took to be an arm—a human arm, swarthy and bare—doubtless the prehensile limb of some intruding robber. The question, as soon as I had recovered power to think, was what to do, and I determined at once to have recourse to a bludgeon with which I had valorously armed myself in view of any such emergency. With this I thought I could, and might under the circumstances, lawfully disable my expected antagonist before he was aware that he had been observed. Getting softly out of bed, I took one step forward and raised my weapon for a blow at what I supposed to be the wrist of the wriggling limb, but before I could execute my fell purpose my terror turned to amazement. The arm suddenly divided into four pieces! four several living entities of the most active kind, each of which took a separate direction across the room. One got up the chimney, another under the bed, and a third, the only one I caught, up the curtains. He was a fine rat, and, I believe, the leader of the burglarious band, and he suffered there and then the just reward of his crime. Looking into the drawer by the keys, I observed, what I had not noticed before, the remains of a *bag* of *biscuits*, which, and not the keys, were evidently the object of their search. I could not understand it then, but I have little doubt now, after some thirty years of cogitating on the matter, that the four disturbers of my peace were acting in concert, and were putting in practice, for the conveyance of biscuits, the plan which, according to your correspondents, they have been known to adopt as to eggs. Else why the extended line which my imagination wrought into an arm?"

A Clever Gymnast.

"*A propos* of the rat, it may be worth while to say that I once saw a rat come up from the hold of a ship and then carefully descend a rope that happened to have been left hanging overboard and reaching down to the water, probably twenty feet below. Arrived at the end of the rope, the rat took a long drink, and then ascended to the deck and scuttled off down below. This was on board an English brig lying at anchor far up the River Plate. There had been no rain for a long time, and I was told the vessel was tight and did not require pumping."

Rats as Egg Stealers.

"I have read the correspondence in *The Times* about rats with the greatest interest, as it has solved a question of theft which had long puzzled me. Some years since, my husband took a house in one of the wildest districts in Wales, for the sake of the shooting. Llwyndwf being sixteen miles from a town, we had to provision accordingly, so I arranged two store rooms for groceries, one for every-day use, the other for occasional. Into that were placed (amongst other things) eggs on a shelf laid in sawdust and dips hung from the shelf, the string from the nail before reaching the dips being nearly three inches long. One morning, on going to the store, I found one pound clean gone, leaving the strings only which had threaded the candles, and there were also two or three eggs missing. I set it down to a two-legged rat; that I had left the key about, and that a little Welsh help we employed was the culprit. However, I said nothing, and I am glad I did not, for the next time the cupboard was visited, another pound of candles was gone, but the short wicks of some were left on the strings, which had been pulled up to the shelf, and laid along it. The weather having been for some days very wet, we could trace the marks of the grease, and there was an egg rolled nearly to the end of the shelf. Evidently the rats had been disturbed, but the mystery always remained how the rats got the eggs off the shelf. At one end of it was a barred window with a wire gauze grating, and the other end terminating with a good fall of some feet to the ground."

(*To be continued.*)

